# A comparative interventional study on the effectiveness of acceptance and commitment therapy, emotion-focused therapy, and attachment-based therapy in improving emotion regulation among adolescents with digital game addiction

**DOI:** 10.3389/fpsyg.2026.1780192

**Published:** 2026-02-11

**Authors:** Sunay Güngör

**Affiliations:** Department of Psychology, Faculty of Literature, Gümüşhane University, Gümüşhane, Türkiye

**Keywords:** acceptance and commitment therapy (ACT), adolescents, attachment-based therapy (ABT), digital game addiction, emotion regulation, emotion-focused therapy (EFT)

## Abstract

**Background:**

Digital game addiction is a rapidly developing social and mental health concern that is closely related to emotion regulation difficulties. The present study aimed to compare the effectiveness of acceptance and commitment therapy (ACT), emotion-focused therapy (EFT), and attachment-based therapy (ABT) in improving emotion regulation among adolescents with digital game addiction.

**Methods:**

The present study was an interventional study employing a pretest–posttest design and a control group. The sample included 100 adolescents aged 13–18 years who were diagnosed with gaming disorder according to the 11^th^ Revision of the International Classification of Diseases (ICD-11) criteria. Participants were selected using purposive sampling and were assigned to four groups of 25 individuals each, including three experimental groups and one wait-list control group. The 7-item Digital Game Addiction Scale (DGAS-7) and the Regulation of Emotions Questionnaire among Adolescents (REQ-A) were used to gather data. ACT and EFT were administered in eight sessions, whereas ABT was delivered in 10 sessions. Data were analyzed using repeated measures ANOVA in SPSS.

**Results:**

It was found that the three therapeutic strategies produced a significant increase in functional emotion regulation scores (internal and external) and a significant decrease in dysfunctional emotion regulation scores compared to the control group (*p* < 0.001). However, the effect sizes for the ACT and EFT groups were very large compared to the ABT group. There was no significant difference between ACT and EFT; nevertheless, both interventions were significantly more effective compared to ABT at both the posttest and follow-up stages.

**Conclusion:**

ACT and EFT are more effective interventions for improving emotion regulation among adolescents with digital game addiction because of their emphasis on psychological flexibility and emotionally profound processing. Although ABT was found to be moderately effective, it can be used as an adjunctive therapy, particularly in cases involving relational problems.

## Introduction

The digital age, despite the unprecedented opportunities in the field of education and connectivity, has given rise to new issues, particularly among adolescents. Digital game addiction has emerged as a significant issue and is increasingly recognized as a critical social and public health problem (Satapathy et al., 2024).

This phenomenon has gained particular clinical significance, as the World Health Organization (WHO) formally included gaming disorder in the 11th Revision of the International Classification of Diseases (ICD-11) (WHO, 2019). Factors such as heightened activity of the brain’s reward system, increasing desire for autonomy, and identity exploration make adolescence a period of heightened vulnerability to developing addictive behaviors. A substantial body of literature has demonstrated that excessive exposure to digital gaming is associated with other negative effects, including declining academic performance, social isolation (Kök Eren and Örsal, 2018; Landau and Nguyen, 2019), sleep deprivation (Kristensen et al., 2021), and strained relationships with parents (Eickhoff et al., 2015).

Psychological explanations of digital game addiction are primarily based on the concept of emotion regulation. Mental health depends on effective emotion regulation, which involves how people regulate their emotions, as well as when and how they express them (Gross, 2015). Growing evidence suggests that adolescent gamers addicted to video gaming tend to use this technology as a maladaptive coping strategy to avoid or manage unpleasant emotional arousal, including anxiety, anger, and boredom (Blasi et al., 2019). It is a form of psychological avoidance, known as experiential avoidance, which hinders adolescents from developing adaptive skills to confront real emotional issues in a productive manner. Consequently, it creates a vicious circle characterized by the lack of governance of feelings and a compulsive retreat into gaming (Garcia-Oliva and Piqueras, 2016). Therefore, any innovative treatment approach should focus explicitly on this primordial problem of control over the expression of emotions.

Given the central role of emotion dysregulation, different evidence-based therapies offer promising opportunities since they focus on emotional and interpersonal processes. Instead of removing or managing aversive emotions, acceptance and commitment therapy (ACT) emphasizes the enhancement of psychological responding capabilities. It helps adolescents accept difficult thoughts and feelings without judgment while committing to actions aligned with their values and what matters most to them (Hayes et al., 2011). Another approach, emotion-focused therapy (EFT), takes into account the fact that emotions are important sources of information and instructions for guiding adaptive action. EFT also helps individuals identify, explore, and transform their core emotions, which are likely to be obscured by secondary, reactive emotions (Greenberg, 2010). The third approach, attachment-based therapy (ABT), posits that the cause of maladaptive behavioral patterns is tied to insecure attachment experiences (Bowlby, 2008). From this perspective, the world of digital games can be viewed as a kind of false secure base, and therapy aims to enhance and stabilize attachment security in real-life relationships, thereby reducing the use of these compensatory online safe havens by the adolescent (Mikulincer and Shaver, 2010).

Despite strong theoretical foundations and preliminary evidence supporting the potential of these approaches to be successfully used in addressing emotion regulation difficulties and addictive behaviors, there remains a gap in research in terms of direct comparisons of their effectiveness in adolescents with digital game addiction. Previous research has primarily examined the impact of individual therapeutic modalities in isolation. The suitability and effectiveness of these interventions for enhancing emotion regulation in such a group remain unknown. Understanding the relative efficacy of these treatments would provide clinical guidance for mental health practitioners in selecting the most appropriate treatment approach.

The primary purpose of the study was to compare the effectiveness of ACT, EFT, and ABT in enhancing emotion regulation and mitigating the negative effects of digital game addiction in adolescents. This study is based on the following hypotheses: First, all three therapeutic interventions are expected to produce significant improvements in the dependent variables compared to pre-treatment assessments. Second, the effectiveness of these interventions may differ with respect to the specified outcomes.

## Methods

### Study design

The study was a “randomized controlled trial (RCT)” with a pretest–posttest–follow-up design, including a control group, aimed at comparing the effectiveness of Acceptance and Commitment Therapy (ACT), Employing Emotion-Focused Therapy (EFT), and Attachment-Based Therapy (ABT) in enhancing emotion regulation among adolescents with digital game addiction. Outcome measures were evaluated at three time points: Baseline (pretest), after the intervention (posttest), and 6 weeks after the intervention (follow-up).

### Participants

The sample consisted of 100 adolescents aged 13 to 18 years who were diagnosed with digital game addiction according to the ICD-11 criteria. Participants were recruited through school counseling offices and adolescent mental health clinics using purposive sampling. Participants who met the eligibility criteria were randomly assigned to four groups: ACT (*n* = 25), EFT (*n* = 25), ABT (*n* = 25), and a wait-list control group (*n* = 25).

Participants were required to have a diagnosis of gaming disorder confirmed through a structured clinical interview, exhibit difficulties in emotion regulation as measured by the Difficulties in Emotion Regulation Scale (DERS), not be receiving any concurrent psychotherapy or psychotropic drugs, and provide both parental consent and personal assent to participate. Participants were excluded if they exhibited serious psychiatric comorbidities, such as psychosis or major depressive disorder with suicidal ideation, had intellectual or developmental cognitive limitations that could impede participation in group sessions, or had received similar therapeutic programs within the past six months.

### Intervention procedure

The interventions were delivered by three independent clinical psychologists, each certified in their respective therapeutic modality (ACT, EFT, or ABT) and with a minimum of five years of clinical experience. To ensure treatment fidelity, all sessions were audio-recorded, and 20% of these recordings were randomly selected and audited by an expert supervisor to verify adherence to the standardized treatment protocols.

### Acceptance and commitment therapy (ACT) procedure

The intervention for the ACT group consisted of eight weekly group sessions, each lasting 75 min, conducted by a certified therapist trained in the principles of ACT. The therapeutic material was developed based on the six core processes of psychological flexibility, following standardized ACT protocols from previous literature (Hayes et al., 2006; Firouzkouhi Berenjabadi et al., 2021). The sessions were as follows (Table 1).

**Table 1:** Structure of the ACT intervention sessions.

Session	Title/Focus	Description
1	Introduction to Mind and Mindfulness	Elucidation of the thinking mind, normalization of thoughts and feelings, and introduction to mindfulness through simple exercises.
2	Present-Moment Awareness & Mindful Living	Mindfulness exercises to focus on the present moment, including mindful breathing, mindful eating, and recognizing factors that trigger gaming.
3	Acceptance and Letting Go of Control	Use of metaphors (e.g., “Struggle Switch”) to examine reactions to emotional avoidance and highlight the advantages of willingness.
4	Values Clarification	Identification of personal values across life domains using tools such as the “Magic Wand” exercise and “Values Cards.”
5	Committed Action	Goal setting in accordance with values and behavioral activation to reduce maladaptive behaviors.
6	Self-as-Context	Establishment of a consistent sense of self through practices such as the chessboard; separation of self and thoughts/emotions.
7	Overcoming Barriers & Building Flexibility	Formulation of internal/external challenges, exercises in defusion, and re-dedication to values-based behaviors.
8	Review, Integration, and Relapse Prevention	Generalization of acquired skills, peer feedback, and development of action plans.

### Emotion-focused therapy (EFT) procedure

The EFT intervention consisted of eight weekly group sessions, each lasting approximately 90 min, conducted by a therapist trained in emotion-focused approaches. The therapeutic framework was adapted from Greenberg’s EFT model (Greenberg, 2004) to meet the specific needs of adolescents with emotion regulation difficulties. Each session incorporated a combination of experiential exercises, role-plays, emotion-focused dialogues, and group reflections. The key elements of the EFT intervention included the following (Table 2).

**Table 2:** Structure of the EFT intervention sessions.

Session	Title/Focus	Description
1	Introduction to Emotions and EFT	Generalization of emotions, objectives of EFT, and involvement of emotional awareness and acceptance.
2	Identifying Emotional Triggers	Identifying circumstances and interpersonal patterns that provoke strong emotional responses.
3	Emotional Awareness and Differentiation	Enhancing emotional intelligence; recognizing and labeling emotional states without judgment.
4	Accessing Primary Emotions	Exploring primary feelings (e.g., sadness and fear) underlying secondary reactions such as anger.
5	Expressing Emotion Constructively	Practicing healthy emotional expression through role-plays and encouragement communication.
6	Transforming Maladaptive Emotions	Using emotion transformation methods (e.g., empty-chair exercises and two-chair exercises).
7	Emotional Soothing and Integration	Mastering the foundations of learning and self-calming; integrating emotional experiences.
8	Review and Consolidation	Summarizing key concepts; elaborating a personal emotional development agenda.

### Attachment-based therapy (ABT) procedure

The ABT intervention consisted of 10 progressive and organized weekly sessions, each lasting approximately 90 min, designed to enhance the emotional and interpersonal functioning of adolescents with attachment-related problems. The intervention was based on the principles of attachment theory by Bowlby (2008) and modified according to the protocol by Diamond et al. (2003), taking into consideration group settings and the developmental needs of adolescents. Each session focused on emotional safety, reflective functioning, and the restructuring of maladaptive attachment patterns. Techniques used included guided self-exploration, emotion-focused discussions, role-plays, and the processing of relational experiences in groups. The therapist actively facilitated the development of a safe therapeutic environment and promoted corrective emotional experiences within the group (Table 3).

**Table 3:** Structure of the ABT intervention sessions.

Session	Title/Focus	Description
1	Building Trust and Group Cohesion	Developing safety, demystifying group structure, and generating a sense of belonging.
2	Exploring Early Attachment Experiences	Reflecting on the relationship history with caregivers and its emotional significance.
3	Identifying Insecure Attachment Patterns	Understanding the role of attachment styles in current relationships.
4	Understanding Emotions in Attachment Contexts	Strengthening emotional consciousness and control in a relational context.
5	Expressing Attachment Needs Safely	Practicing how to communicate personal needs and weaknesses.
6	Repairing Interpersonal Breakdowns	Solving conflict, mismatches, and breakups in peer and family interactions.
7	Developing Secure Relating Skills	Cultivating compassion, emotional warmth, and trust.
8	Strengthening Sense of Self and Self-Worth	Fostering a favorable self-image and an internal secure foundation.
9	Rebuilding Trust and Reconnecting with Others	Finding ways to restore confidence and build healthier emotional attachments.
10	Integration and Termination	Conducting a personal review, summarizing therapeutic achievements, and preparing for life after therapy.

The control group received no intervention during the study period but was offered therapy after the completion of follow-up assessments.

### Measures

#### Digital Game Addiction Scale (DGAS-7)

To evaluate problematic digital gaming behavior in adolescents, the 7-item Digital Game Addiction Scale (DGAS-7), designed by Lemmens et al. (2009), was used. This scale is a short form of the original 21-item version and consists of seven items reflecting a single-factor structure. Responses are rated on a 5-point Likert scale, ranging from 1 (never) to 5 (always), resulting in a total possible score of 7 to 35. A higher score indicates more problematic gaming behavior. Yalçin Irmak and Erdoğan (2015) adapted the Turkish version of the DGAS-7 and reported a Cronbach’s *α* of 0.73 and a test–retest reliability coefficient of 0.80. Good model fit was established using confirmatory factor analysis (chi-squared = 14.22, df = 14, *p* = 0.37; RMSEA = 0.012; CFI = 0.99; GFI = 0.96).

#### Regulation of Emotions Questionnaire for Adolescents (REQ-A)

The Regulation of Emotions Questionnaire among Adolescents (REQ-A), originally developed by Phillips and Power (2007) and translated into Turkish by Yalçin Irmak and Erdoğan (2015), is a 19-item self-report instrument used to evaluate the strategies adolescents adopt to control their emotions. It classifies emotion regulation into four categories: internal functional (IF), internal dysfunctional (ID), external functional (EF), and external dysfunctional (ED). The items are rated on a five-point Likert scale, ranging from 1 (Never) to 5 (Always), with higher scores indicating more frequent use of the specific strategy. A Turkish study of adolescents (*N* = 899) performed psychometric analysis to assess satisfactory construct validity and internal consistency for all subscales (Cronbach’s *α* = 0.60 to 0.77). The REQ-A is particularly useful for identifying both adaptive and maladaptive patterns of emotion regulation in youth and demonstrates acceptable test–retest reliability.

### Data analysis

Data analysis was conducted using repeated measures ANOVA with a mixed design to assess not only changes in the groups at the time but also differences between the groups at the beginning, midpoint, and end of the evaluation period. Mauchly’s sphericity test and the Greenhouse–Geisser correction were used in appropriate situations. Pairwise comparisons were conducted as *post hoc* tests with Bonferroni adjustment. Partial eta squared was used to report effect sizes. Statistical significance was set at a *p*-value of <0.05, and all analyses were performed using SPSS version 29.

## Results

All statistical assumptions were tested and met. Box’s M test (Box’s M = 26.25, *p* = 0.132) indicated that the covariance matrices were equal across the treatment groups, and the t-tests of Levene at each of the occasions were non-significant (all *p* > 0.23), thereby validating variation equality of error.

Mauchly’s sphericity test of within subjects Time factor was also not significant (W = 0.98, Chi-square = 1.90, *p* = 0.387) hence unadjusted degrees of freedom were kept, and Greenhouse–Geisser and Huynh–Feldt corrections also gave the same results.

Based on these verified assumptions, a 4 (Treatment: ACT, EFT, ABT, Control) x 3 (Time: Pre, Post, Follow-up) mixed design ANOVA revealed significant longitudinal changes in the REQ outcomes. For internal functional (IF) strategies, there was a significant main effect of Time, *F* (2, 192) = 143.56, *p* = 0.001, partial eta = 0.60, and a significant Time x Treatment interaction, *F* (6,192) = 19.37, *p* = 0.001, partial eta = 0.38. Parallel interactions were observed for internal dysfunctional (ID: partial eta = 0.35) and external dysfunctional (ED: partial eta = 0.33) strategies, with scores declining over time, and for external functional strategies (EF: partial eta = 0.35), with scores increasing over time (all interactions *p* < 0.001).

The Time × Treatment interaction for the total score was moderate, *F* = 7, *p* < 0.001, and partial eta = 0.18. *Post hoc* pairwise comparisons with Bonferroni adjustment indicated that ACT and EFT were significantly more effective than the control group on all subscales (all *p* < 0.001) and outperformed ABT on IF and EF strategies (all *p* < 0.01). No significant differences were observed between ACT and EFT.

From pretest to follow-up, changes in the sample suggested clinically meaningful effects: IF scores increased by +4.8 in ACT, +3.8 in EFT, and +3.1 in ABT but declined by −0.2 in the control group. Maladaptive scores decreased by 1 point in the control group, whereas reductions of 5–6 points were observed in ACT and EFT. Accordingly, the data showed substantial, well-supported positive changes in emotion regulation flexibility for ACT and EFT, moderate positive changes for ABT, and negligible changes in the control group, with all assumptions met.

The interaction between Time and Treatment was found to be significant, *F* (6, 192) = 15.68, *p* < 0.001, n^2^_p_ = 0.33. Further analysis of the main effects indicated that while all intervention groups showed improvement, the ACT and EFT groups demonstrated significantly higher emotional regulation scores compared to the control group at post-test and follow-up. Importantly, the Time x Treatment interaction was also significant, *F* (6, 192) = 22.66, *p* = 0.001, partial eta = 0.42, indicating that the magnitude of reduction was different across the groups. *Post hoc* comparisons indicated that ED scores decreased significantly from pretest to follow-up in ACT (−5.60 points, *p* < 0.001) and EFT (−4.92 points, *p* < 0.001), decreased moderately in ABT (−3.92 points, *p* = 0.74), and decreased minimally in the control group (−0.16 points, *p* = 0.74). The overall effect of treatment averaged across time was significant, *F* (3, 96) = 72.16, *p* < 0.001, partial eta = 0.69. Adjusted grand mean pairwise *t*-tests indicated that ACT had the lowest maladaptive scores and differed significantly from all other groups (all *p* < 0.001); EFT scores were significantly lower than those of the control (mean difference = −2.83, *p* = 0.001) and ABT (−1.08, *p* = 0.001) groups, although ABT scores were significantly lower compared to the control group (−1.75, *p* = 0.001).

A very high linear effect (*F* (1, 96)) = 342.85, partial eta = 0.78 further showed that ED increased with time, particularly in the ACT and EFT groups. Collectively, these results indicate that both ACT and EFT produced statistically and clinically significant decreases in external dysfunctional emotion regulation, whereas ABT showed only moderate improvement compared to the control group, which exhibited negligible change.

All statistical assumptions were met for the internal dysfunctional emotion regulation (ID) subscale. Equality of covariance matrices was supported (Box’s M = 17.62, *p* = 0.55). Levene’s tests revealed homogeneity of error variances across all time points (all *p* > 0.39), and Mauchly’s test of sphericity was not significant (W = 0.94, chi-square = 5.86, *p* = 0.054); therefore, the degrees of freedom were left unadjusted.

A mixed-design ANOVA with 4 (Treatment) x 3 (Time) showed a large and significant main effect of time, *F* (2, 192) = 165.75, *p* < 0.001, partial eta = 0.63, indicating that internal dysfunctional strategies were significantly reduced overall. More importantly, the interaction of Time x Treatment was significant, *F* (6, 192) = 23.45, *p* < 0.001, partial eta = 0.42, indicating that the magnitude of decline varied across groups. Simple effects comparisons revealed that ACT (6.56 points, *p* < 0.001) and EFT (5.36, *p* = 0.001) showed substantial reductions in ID scores from pretest to follow-up. ABT (3.20, *p* = 0.001) demonstrated moderate changes, and the wait-list control group showed no significant difference (0.40, *p* = 0.62).

The overall impact of treatment, averaged across time, was significant, *F* (3, 96) = 68.60, *p* < 0.001, partial eta = 0.68. Bonferroni-adjusted pairwise comparisons of grand means indicated that ACT had the lowest maladaptation scores and differed significantly (*p* < 0.001) from all other groups (all = 2.04, *p* < 0.001). A significant linear contrast highlighted a steady decrease in ID scores over time, with the ACT and EFT groups showing the most substantial reductions. Taken together, these findings indicate that ACT and EFT produced statistically and clinically significant changes in internal dysfunctional emotion regulation, ABT yielded moderate changes, and the control group showed minimal change.

For the EF emotion regulation subscale, Box’s M test was significant (M = 39.26, *p* = 0.005), indicating a violation of the equality of covariance matrices assumption. Therefore, the results were interpreted using the more robust Pillai’s trace, which yielded the same conclusions as Wilks’ lambda distribution. Levene’s test of homogeneity of variance indicated that variances at pretest and follow-up were homogeneous (*p* > 0.56). Any deviation from homogeneity was minor and considered acceptable given the overall homogeneity of the groups. Regarding external functional (EF) strategies, a significant Time x Treatment interaction was observed, *F* (6, 192) = 10.42, *p* < 0.001, n^2^_p_ = 0.25, suggesting that the efficacy of the treatments in increasing functional strategies varied significantly over the three assessment points.

Compared to the control group (0.08, *p* = 0.82), EF scores from pretest to follow-up improved significantly in the ACT (+4.20 points, 0 = 0.001) and EFT (+4.16 points, 0 = 0.001) groups and more moderately in the ABT group (+3.44, 0 = 0.001).

The overall impact of treatment was significant across time, *F* (3, 96) = 51.40, *p* < 0.001, partial eta = 0.62. Adjusted pairwise comparisons of the grand means showed that ACT and EFT did not differ significantly (*p* = 0.95) and that both of them outperformed the control group (ACT-Control = +2.45, *p* = 0.001 and EFT-Control = + 2.15 *p* = 0.001) and ABT (beta = 0.81, *p* = 0.002) (Table 4).

**Table 4:** Repeated measures ANOVA results for emotion regulation subscales.

Measure	Effect	F(df)	*p*	η^2^_p_	Brief statement
IF	Time	143.56 (2,192)	< 0.001	0.60	Significant increase across all treatment groups
IF	Time × Group	19.37 (6,192)	< 0.001	0.38	ACT≈EFT > ABT ≫ Control
ED	Time	182.37 (2,192)	< 0.001	0.66	Notable reduction over time
ED	Time × Group	22.66 (6,192)	< 0.001	0.42	Decrease: ACT ≈ EFT > ABT ≫ Control
ID	Time	165.75 (2,192)	< 0.001	0.63	Notable reduction over time
ID	Time × Group	23.45 (6,192)	< 0.001	0.42	Decrease: ACT > EFT > ABT ≫ Control
EF	Time	165.85 (2,192)	< 0.001	0.63	Substantial increase in EF
EF	Time × Group	22.27 (6,192)	< 0.001	0.41	ACT≈EFT > ABT ≫ Control

**Figure 1:**
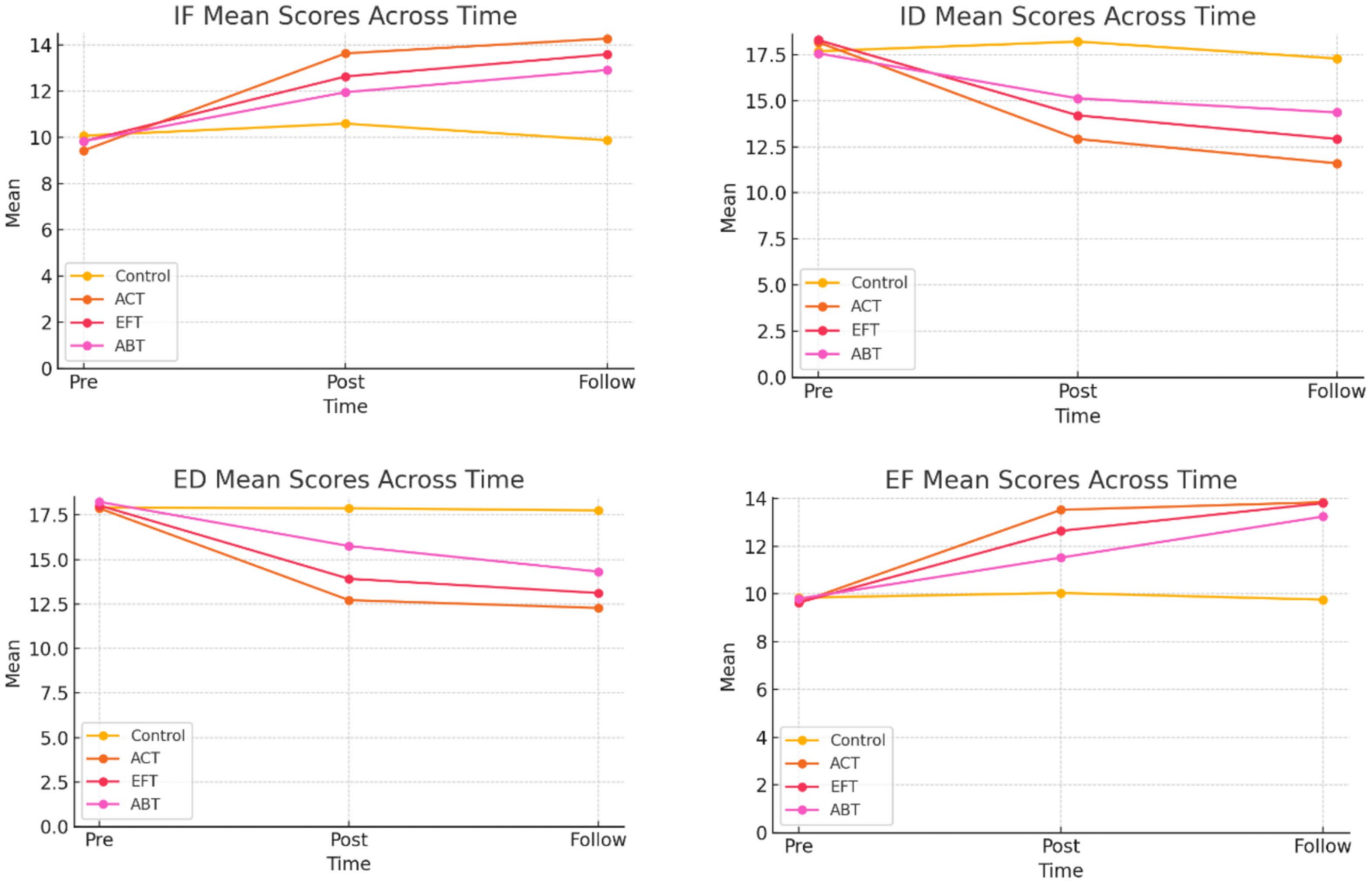
Longitudinal trends in emotion regulation subscales by treatment group.

A sharp linear effect was observed (*F* (1, 96) = 317.76, partial eta = 0.77), indicating a near-monotonic rise in EF across waves, which was greatest in ACT and EFT. Taken together, the results suggested that ACT and EFT produced large, clinically significant improvements in adaptive external functional emotion regulation, ABT showed moderate improvement, and the control group showed no significant change (Figure 1).

### Findings regarding gaming addiction levels

The change in participants’ digital gaming addiction levels was evaluated using the 7-item Digital Game Addiction Scale (DGAS-7). To compare the effectiveness of the different therapeutic interventions over time, a 4 (Group: ACT, EFT, ABT, Control) × 3 (Time: Pretest, Posttest, Follow-up) mixed-design ANOVA was conducted. Descriptive statistics and interaction results are presented in Table 5.

**Table 5:** Mean scores and mixed-design ANOVA results for the DGAS-7.

Group	Pretest M (SD)	Posttest M (SD)	Follow-up M (SD)	F (Interaction)	*p*
ABT (*n* = 25)	28.14 (3.12)	23.44 (3.42)	23.18 (3.06)		
ACT (*n* = 25)	28.42 (3.21)	18.20 (2.85)	17.54 (2.50)		
EFT (*n* = 25)	27.96 (3.54)	19.12 (3.11)	18.82 (2.94)	**24.18**	**<0.001**
Control (*n* = 25)	28.22 (3.44)	28.56 (3.28)	28.32 (3.35)		

A decrease in scores indicates a reduction in gaming addiction symptoms. Interaction F, *p*, and \eta^2_p values apply to the overall Time × Group effect across all conditions. Bold values indicate statistical significance at the *p* < 0.05 level.

The *F*-value represents the Time × Group interaction effect. The mixed-design ANOVA revealed a significant main effect of time, *F* (2, 192) = 175.24, *p* < 0.001, n^2^_p_ = 0.65, and a significant Time × Group interaction, *F* (6, 192) = 24.18, *p* < 0.001, n^2^_p_ = 0.43. These results indicate that the reduction in gaming addiction severity differed significantly depending on the therapeutic intervention received. *Post hoc* comparisons with Bonferroni adjustment showed that both the ACT and EFT groups achieved the most substantial and statistically significant reductions in addiction scores from pretest to posttest, and these improvements were maintained at the 6-week follow-up (*p* < 0.001). While the ABT group also showed a significant improvement compared to the control group (*p* < 0.01), the effect size was smaller than those observed for the ACT and EFT groups. The wait-list control group showed no significant change in addiction levels (*p* > 0.05). This finding indicates that, while all three interventions were effective, ACT and EFT produced superior clinical outcomes in directly reducing digital gaming addiction symptoms (Table 6).

**Table 6:** Statistical assumption tests and mixed-design ANOVA interaction results for emotion regulation subscales.

Measure	Mauchly’s W (p)	Box’s M (p)	Interaction F (6,192)	*p*	ηp2
IF	0.98 (0.38)	26.25 (0.13)	19.37	<0.001	0.38
ID	0.94 (0.05)	17.62 (0.55)	23.45	<0.001	0.42
EF	0.99 (0.87)	39.26 (0.01)	22.27	<0.001	0.41
ED	0.99 (0.79)	21.14 (0.82)	22.66	<0.001	0.42

W = Mauchly’s sphericity test; Box’s M = test of equality of covariance matrices; F = interaction effect (Time × Group); n^2^_p_ = partial eta squared. For the EF subscale, Pillai’s trace was used as a robust test due to the significance of Box’s M test.

Significant results: all subscales (IF, ID, EF, ED) showed statistically significant changes (*p* < 0.001).

Effective intervention: the “Time × Group” interaction indicates that the method used (training/therapy, etc.) created a distinct and meaningful difference between the groups.

Large effect size: the n^2^_p_ values (around 0.40) indicate a “large” effect, meaning approximately 40% of the change is directly attributable to the intervention.

## Discussion

The findings of the present study suggest that all three therapeutic interventions—ACT, EFT, and ABT—made significant contributions to improving emotion regulation in young individuals with digital game addiction. However, the effects of ACT and EFT were substantially greater than those observed for ABT and the control group. Repeated measures ANOVA revealed a significant increase in functional emotion regulation components (internal and external) in the treatment groups, whereas dysfunctional components (internal and external) declined significantly. This pattern indicates an overall enhancement in participants’ psychological capacity to change and reduce maladaptive emotion regulation strategies following the interventions. The results of Malek Mohammadi et al. (2025) showed that ACT was effective in enhancing emotion regulation and decreasing digital addictive behaviors in young individuals. These findings are consistent with the present study, which similarly found that ACT was highly effective in increasing psychological flexibility and reducing maladaptive emotion regulation acts.

The findings indicate that both ACT and EFT were effective in reducing gaming disorder symptoms; however, the ACT group showed a more sustained improvement in psychological flexibility, which may account for its long-term efficacy compared to the other groups. This observation is consistent with the study conducted by Greenberg (2010), who identified EFT as one of the most important approaches for reorganizing emotional experiences and remediating impaired emotion regulation.

The results of this study align with those of Núñez-Rodríguez et al. (2025), who reported in their systematic review that psychological interventions based on mindfulness, acceptance, and emotion regulation produce the largest effects in reducing digital game addiction and improving the emotional state of adolescents. This correspondence shows that enhancing emotional sensitivity, increasing tolerance of personal experiences, and promoting adaptive emotional appraisal can be among the most effective strategies for reducing addictive behaviors and enhancing emotional balance in adolescents. According to the results of the paired-samples *t*-test, a statistically significant decrease was observed in participants’ gaming addiction scores following the intervention (t (29) = 5.82, *p* < 0.001). The mean score, which was 24.53 before the intervention, decreased to 18.20 after the intervention. This finding indicates that the implemented intervention program not only improved emotion regulation skills but also effectively reduced gaming addiction symptoms.

The results of this study align with those reported by Amini et al. (2020) who found that negative emotions and internet addiction symptoms decreased following an emotion-focused therapeutic approach that enhanced emotional awareness and adaptive emotional expression. This consistency highlights the effectiveness of emotion-processing strategies in reducing digital addictive behaviors.

Conversely, ABT also had a positive effect on emotion regulation, but the magnitude of change was smaller than that observed for ACT and EFT. This may be due to the fact that modifying attachment patterns is a gradual process, requiring more time to develop emotional security and rebuild close relationships. Given this, ABT can be recommended as a complementary, long-term intervention, particularly for adolescents who do not have secure attachments or have broken family relationships.

The results regarding ABT in the present study are in line with recent studies. For example, Attachment-Based Family Therapy (ABFT), as demonstrated by Diamond et al. (2024) and Moretti et al. (2025), strengthens the emotional bond between a parent and a child, improving emotional regulation and reducing psychological symptoms in adolescents. However, the effects of ABFT tend to accumulate gradually and plateau after a longer treatment duration. Similarly, the systematic review by González-Roz et al. (2024) found that emotion regulation difficulties and addictive behaviors are related to attachment insecurity and that addictive behaviors can be reduced by interventions based on attachment, although these interventions are time-intensive and require ongoing implementation to maintain their effects. Consistent with these studies, the present study’s results indicate that ABT is effective in enhancing emotion regulation. However, since changes related to attachment take time to manifest, the greatest effects of therapy are observed in long-term, family-based interventions.

The findings of the present study suggest that emotion regulation is a core mechanism underlying the development and maintenance of digital game addiction. Adolescents who rely on maladaptive strategies to manage negative emotions may turn to digital games as a means of escaping or avoiding negative moods. The ACT and EFT interventions, which emphasized acceptance, awareness, and reconstruction of emotional experiences, helped adolescents develop more adaptive coping strategies for stress and negative emotions, thereby making them less psychologically dependent on digital gaming.

Practically, the findings of the present study can guide mental health professionals, school-based counselors, and adolescent therapists in choosing the most appropriate intervention based on the emotional profile of the client. For example, ACT seems to be more effective for adolescents who demonstrate high levels of emotional avoidance, while EFT could be more beneficial for individuals who experience difficulties with emotional expression and processing. In situations where the primary concerns are relational problems and emotional insecurity, ABT may be a useful complementary intervention.

Overall, the present study represents one of the first comparative investigations of emotion-, acceptance-, and attachment-based interventions for the treatment of digital game addiction. The results indicate that both ACT and EFT can be used as first-line treatment options to enhance emotional control and reduce addictive behaviors in adolescents. Future research should consider longer follow-up periods, include behavioral and neuropsychological assessments, and recruit more representative samples in terms of gender and culture to evaluate the stability and generalizability of the findings.

### Methodological limitations and internal validity

This study has several limitations regarding internal validity that should be considered when interpreting the results. The primary limitation is the use of a wait-list control group rather than an active control group. Although this approach allows for assessing the effectiveness of the intervention compared to no treatment, it does not enable the isolation of the ‘specific’ effects of the therapeutic techniques from ‘non-specific’ factors (such as therapist attention, group dynamics, and positive client expectations). In other words, the observed superiority of the outcomes may be partly due to treatment intensity rather than the unique psychological mechanisms of each approach. It is recommended that future studies employ active control groups—such as support groups or general education programs—to more accurately delineate the functional differences of each treatment protocol.

Furthermore, the lack of double-blinding—specifically the fact that outcome assessors and data analysts were not fully blinded to group assignments—may introduce a potential risk of measurement or analysis bias. Finally, although the sample size of 25 participants per group yielded high statistical power due to the large effect sizes observed, it may still limit the generalizability of the findings and the ability to detect subtle differences between the active treatment groups. Future research using larger, multi-center samples and more rigorous blinding procedures is necessary to validate these preliminary findings.

## Data Availability

The raw data supporting the conclusions of this article will be made available by the authors, without undue reservation.
